# Earlier Initiation and Use of a Greater Number of Iron-Folic Acid Supplements during Pregnancy Prevents Early Neonatal Deaths in Nepal and Pakistan

**DOI:** 10.1371/journal.pone.0112446

**Published:** 2014-11-14

**Authors:** Yasir Bin Nisar, Michael J. Dibley

**Affiliations:** Sydney School of Public Health, The University of Sydney, Australia; The Hospital for Sick Children, Pakistan

## Abstract

**Introduction:**

Early neonatal deaths account for 75% of neonatal deaths globally. Antenatal iron-folic acid (IFA) supplementation has significantly reduced the risk of early neonatal deaths in China and Indonesia. We investigated the impact of antenatal IFA supplements on the risk of early neonatal deaths in Nepal and Pakistan during the last decade.

**Methods:**

Data from the most recent singleton live-births of 8,186 from two Nepal Demographic and Health Surveys (DHS) and 13,034 from two Pakistan DHS were selected for the current study. The primary outcome was early neonatal deaths and the main study variable was antenatal IFA supplementation. Analyses used multivariate Cox proportional regression, adjusted for the cluster sampling design and for 18 potential confounders.

**Findings:**

The adjusted risk of early neonatal deaths was significantly reduced by 51% (aHR = 0.49, 95% CI = 0.32–0.75) in Nepal and 23% (aHR = 0.77, 95% CI = 0.59–0.99) in Pakistan with any use of IFA compared to none. When IFA supplementation started at or before the 5^th^ month of pregnancy, the adjusted risk of early neonatal mortality was significantly reduced by 53% in Nepal, and 28% in Pakistan, compared to no IFA. When >90 IFA supplements were used and started at or before 5^th^ months, the adjusted risk of early neonatal deaths was significantly reduced by 57% in Nepal, and 45% in Pakistan. In Nepal 4,600 and in Pakistan 75,000 early neonatal deaths could be prevented annually if all pregnant women used >90 IFA supplements and started at or before the 5^th^ month of pregnancy.

**Conclusions:**

Any use of IFA supplements was significantly associated with reduced risk of early neonatal deaths in Nepal and Pakistan. The greatest mortality sparing effect of IFA on early neonatal deaths in both countries was with early initiation and use of a greater number of supplements.

## Introduction

Globally, of 6.6 million under-five deaths, 2.9 million were neonatal deaths accounting for 44% of the under-five deaths in 2012 [1]. Three-quarters of neonatal deaths globally occur in the first week of life, known as the early neonatal deaths. [2], [3] Most of the neonatal deaths (99%) arise in low-middle income countries [2]. In Pakistan and in Nepal, neonatal deaths account for 49% and 58%, respectively, of under-five deaths [1]. The fourth Millennium Development Goal (MDG-4) aims to reduce under-five deaths by two-thirds by 2015 [1]. Therefore, to achieve national targets for MDG-4 in many developing countries, it is important to reduce deaths during the neonatal period.

Preterm birth complications and birth asphyxia are the most common causes of early neonatal deaths [3]. Maternal anaemia in the first or second trimester of pregnancy is associated with a substantially higher risk of low birth weight and preterm birth deliveries [4]. In South Asia, it is estimated that 52% of women are anaemic at some stage during pregnancy [5], and about half of this burden is assumed to be due to iron deficiency [6]. Daily use of antenatal iron-folic acid (IFA) supplements significantly reduces the prevalence of maternal anaemia and risk of low birth weight [4], [7]. The World Health Organization (WHO) guidelines, therefore, recommend distribution of IFA supplements to all pregnant women, as a part of antenatal care services programs. Further, where the prevalence of anaemia in pregnancy is over 40%, a daily dose of 60 mg of elemental iron is preferred over a lower dose of 30 mg [8].

In Nepal and Pakistan IFA supplements are distributed to pregnant women through the public sector health facilities and by community health worker programs [9]–[11]. However, the coverage of antenatal IFA supplements used in Pakistan has remained static during the last decade [9], [10]. The latest Pakistan Demographic and Health Survey (PDHS) 2012–13 reported that 45% of pregnant women took any IFA supplements in their most recent pregnancy within 5 years prior to the survey, and only 22% took ≥90 supplements throughout pregnancy [9]. In contrast, Nepal has shown a substantial increase in the coverage of use of antenatal IFA supplements during last decade primarily due to modification of their existing IFA supplements program [11]–[13]. The latest Nepal Demographic and Health Survey (NDHS) 2011 reported that 80% of pregnant women took any IFA supplements during their most recent pregnancy within 5 years prior to the survey and 56% took ≥90 supplements throughout pregnancy [11]. Studies have reported a significant reduction in the risk of early neonatal and neonatal deaths with use of any antenatal IFA supplements [14]–[16]. A few studies have also reported an effect of antenatal IFA supplements on childhood mortality [15], [17]. The overall aim of the current study was to investigate the effect of use of IFA supplements during pregnancy on the risk of early neonatal deaths in Nepal and Pakistan during the last decade.

## Methods

### Data sources

We used the information of most recent singleton live-births from two NDHS, 2006 [12] and the 2011 [11], and from two PDHS, 2006–07 [10] and the 2012–13 [9], in this analysis. These household surveys have collected information from nationally-representative samples for demographic, health, and nutrition indicators [18].

The details of the sampling technique used in these surveys have been described elsewhere [18]. Briefly, a multistage, stratified, cluster random sampling was used in each survey to collect demographic and health information by conducting interviews with ever-married women in reproductive age (15–49 years). On average, the response rate was over 98% in the NDHS [11], [12], and over 93% in the PDHS [9], [10]. The complete birth history of the respondents was ascertained, and for the most recent live-birth within 5 years prior to interview, additional information about the use of antenatal care, delivery and postnatal care services was recorded. The birth history listed all live-births of a respondent in a chronologic order, and included the date of the birth of each child, the singleton or multiple status, child's sex, the survival status of the child on the day of the interview, and if deceased, the date of death [9]–[12].

We used the survival information of 4,145 and 4,051 most recent singleton live-births within 5 years prior to interview from the NDHS 2006 and the NDHS 2011, respectively. Similarly, survival information of 5,660 and 7,374 most recent singleton live-births within 5 years prior to interview from the PDHS 2006–07 and the PDHS 2012–13, respectively, were used. Sampling weights were applied to compensate for the multistage cluster sampling design.

### Ethics

Informed verbal consent was obtained from each respondent in the NDHS and the PDHS. The current analysis was approved by the Human Research Ethics Committee of University of Sydney.

### Primary outcome and study variables

The primary outcome was early neonatal death, defined as death within first week of life (0–6 days of life) [19]. The main study variable examined was use of any IFA supplements during the most recent pregnancy in the last 5 years prior to the interview date. A respondent who had a recent live-birth within 5 years prior to the interview was asked the following questions in the survey: “During this pregnancy, were you given or did you buy any iron-folic acid tablets?” and “During the whole pregnancy, for how many days did you take the tablets?” A mother was categorised as using any antenatal IFA supplements if she took supplements for at least a day during her pregnancy. To assess the effect of timing of the start of supplementation on the mortality outcome, the time of first antenatal care examination was considered as a proxy for the start of supplements. As anaemia during first two trimesters of pregnancy is associated with poor pregnancy outcomes [4], therefore, we categorised the timing of start of IFA supplements as ‘at or before 5^th^ month of pregnancy’ and ‘after 5^th^ month of pregnancy’. To evaluate the impact of number of supplements used on mortality outcome, the recorded number of IFA supplements used throughout their pregnancy were categorised as ‘1–90 supplements used’, or ‘ >90 supplements used’. A combined variable was constructed combining the number of IFA supplements used with the timing of the start of supplements. This variable was used to investigate the effect of the timing of the start of supplementation and the total number of supplements used on the mortality outcome, as ‘at or before 5^th^ month of pregnancy and 1–90 supplements used’, ‘at or before 5^th^ month of pregnancy and >90 supplements used’ and ‘after 5^th^ month of pregnancy and any IFA supplements used’. We considered use of >240 supplements as implausible and excluded those cases from the analysis (93 cases from NDHS, and 708 cases from PDHS). Furthermore, if a woman started supplements in the 5^th^, 6^th^, 7^th^, 8^th^, and 9^th^ month of her pregnancy, we considered that the maximum number of supplements that she could have used was 150, 120, 90, 60 and 30 supplements, respectively, assuming once daily uptake of supplements. Therefore, records of women in their 5^th^ to 9^th^ month of pregnancy, who reported exceeding the maximum number of supplements, were also excluded from the analysis, (148 records from NDHS; and 781 records from PDHS).

### Potential confounding factors

The Mosley and Chen framework [20] for child survival was adapted for the current study. We examined 15 potential confounding factors and classified them into three main groups–community-level and socioeconomic determinants; maternal and newborn characteristics; and perinatal health care services variables. We assessed seven community-level and socioeconomic status determinants in the analyses. These included a combination of region/province and area of residence, maternal marital status, maternal level of attained education, maternal occupation, paternal level of attained education, paternal occupation, and pooled household wealth index. We selected five maternal and newborn characteristics: maternal age at childbirth, child’s sex, a combination of birth rank and birth interval, the maternal desire for the pregnancy, maternal perceived birth size, and timing of initiation of breastfeeding. Three perinatal health care variables were assessed, which included the number of antenatal care visits, the place of delivery, and a combined variable for mode of delivery with delivery assistance.

In addition, we considered three other important factors in our analyses. The differences in the recall period were adjusted by constructing a variable as the difference in days between the date of the birth of the child and the date of interview. Similarly, we also adjusted for secular trends in maternal iron status and development of perinatal health services with the year of birth of the child. Both of these variables were retained in all regression models. We also adjusted our analysis for the average cluster coverage of Bacillus Calmette-Guerin (BCG) vaccine, as several studies have reported a protective effect of BCG vaccination on child mortality [21]–[24].

A household wealth index variable was constructed for household economic status by using pooled data and a principal component analysis [25] of household facilities and assets including type of toilet, main material of floor, main material of wall, source of drinking water, availability of electricity, possession of radio, television, fridge, or telephone and bicycle/motorcycle/car. This household wealth index was used to rank households across the two surveys and was divided into quintiles for analysis.

The variables of birth rank and birth interval were combined as a 5-category composite variable that consisted of 1^st^ birth-rank infants, 2^nd^ or 3^rd^ birth-rank, with a birth interval >2 years, 2^nd^ or 3^rd^ birth-rank, with a birth interval ≤2 years, ≥4^th^ birth-rank with a birth interval >2 years, and ≥4 birth-rank with a birth interval ≤2 years.

### Statistical analysis

STATA 13.1 (Stata–Corp, College Station, TX, USA) with ‘svy’ commands to allow for adjustments for the cluster sampling design was used for data analysis. The characteristics of study population and factors that were potentially associated with the primary outcome were examined with frequency tabulations. Cox proportional hazards regression was used to examine associations between study factors and the primary outcome, initially with unadjusted regression analysis for each potential factor and later on multivariate analyses to assess the independent effects of each factor after controlling for the other covariates.

We considered a multi-stage backward elimination modelling technique for the assessment of potential confounding factors and the primary outcome. At the initial stage, community-level and socioeconomic variables were assessed and significant factors associated with the mortality outcome were retained in a model. Next, maternal and newborn characteristics were assessed in a model which contained the significant community-level and socioeconomic determinants for the primary outcome. Then, perinatal health care services variables were assessed in a model which had the significant community-level and socioeconomic determinants and maternal and newborn characteristics for the primary outcome. In the last stage, each study variable was assessed separately in a model which contained the significant community-level and socioeconomic determinants, maternal and newborn characteristics and perinatal health care variables for the mortality outcome. Level of significance was considered as 0.05, unless the variable had been apriori selected for inclusion. Hazard ratios (HRs) and their 95% confidence intervals (CIs) derived from adjusted Cox proportional models were used to investigate the effect of the study factors on the mortality outcome.

Population attributable risks (PAR) were calculated to assess total risk of mortality outcome in the general population that was attributable to women who did not start at or before the 5^th^ month of pregnancy and did not take >90 supplements in Nepal and Pakistan. It was assumed that the association between IFA supplementation and mortality was causal and that removal of IFA supplementation had no effect on the distribution of other factors associated with mortality. The following formula was used to calculate PAR [26]–[28].



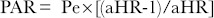



Where, ‘aHR’ was the adjusted hazard ratio for early neonatal deaths of infants whose mother did not start supplements at or before the 5^th^ month of their pregnancy and did not take >90 supplements. ‘Pe’ was the proportion of early neonatal deaths associated with having a mother who did not start supplements at or before the 5^th^ month of their pregnancy and did not use >90 supplements. Using the PAR calculations with annual number of early neonatal deaths [9], [11], and the number of births [29], [30], the number of early neonatal deaths that could be prevented per annum if all pregnant women use >90 supplements starting at or before the 5^th^ month of their pregnancy were estimated separately for Nepal and Pakistan. However, the findings based on PAR estimates depend on the prevalence of the exposure variable, which might vary across populations even within the same country.

## Results

There were 8,196 most recent singleton live-births within 5 years prior to interview, with 147 neonatal and 124 early neonatal deaths in Nepal. In Pakistan, of 13,034 most recent singleton live-births within 5 years prior to the interview date, there were 485 neonatal and 389 early neonatal deaths.

The distribution of community-level and socioeconomic status, maternal and newborn characteristics and perinatal health care services variables of the most recent singleton live-births within 5 years prior to the interview date in Nepal and Pakistan are shown in Table 1. The study population in both countries showed a predominant rural population with low maternal and paternal educational status. In Nepal, nearly a quarter of women were not working for the last 12 months prior to the interview date while in Pakistan nearly three-quarters of the women were not working. Teenage pregnancies were common in both countries with 55% of the Nepalese and 41% of the Pakistani women were <20 years of age at the time of childbirth. About 17% of the Nepalese and 25% of the Pakistani infants had smallest or smaller than average birth size. In Nepal 64% mothers reported to start breastfeeding within 1 hour of delivery whereas in Pakistan only 36% of mothers did so. About 40% of Nepalese mothers and 33% of Pakistani mothers had ≥4 antenatal care visits during their last pregnancy within 5 years prior to interview. In Nepal 70% and in Pakistan 50% of mothers were delivered by traditional birth attendants or by other untrained persons. Home deliveries were common in both countries, as nearly 70% of Nepalese mothers and 55% of Pakistani mothers were delivered at home during their most recent pregnancy within 5 years prior to interview.

**Table 1:** Prevalence of community-level and socioeconomic status, maternal and newborn characteristics, and perinatal health care service determinants of singleton live-births within 5 years prior to interview in Nepal and Pakistan.

Variable	Nepal Pooled NDHS (2001, 2006 and 2011)			Pakistan Pooled PDHS (2006–07 and 2012–13)		
	n†	n††	%††	n†	n††	%††
**Community-level and socioeconomic determinants**						
**Region and area of residence**						
**Nepal**				**NA**		
Terai-urban	1028	454	5.6			
Terai-rural	2573	3731	45.8			
Hill-urban	737	483	5.9			
Hill-rural	2518	2837	34.8			
Mountain-urban	115	12	0.2			
Mountain rural	1225	630	7.7			
**Pakistan**	NA					
Punjab*-urban				2185	2187	16.9
Punjab*-rural				3247	5183	39.9
Sindh-urban				1424	1297	10.0
Sindh-rural				1753	1783	13.7
Khyber Pakhtunkhwa-urban				854	298	2.3
Khyber Pakhtunkhwa-rural				1756	1623	12.5
Balochistan-urban				745	121	0.9
Balochistan-rural				1070	487	3.8
**Maternal marital status**						
Currently Married	8093	8043	98.7	12,890	12805	98.7
Formerly Married	103	105	1.3	144	175	1.4
**Maternal educational status**						
Secondary and above	2467	2441	30.0	3,353	3176	24.5
Completed primary	1547	1563	19.2	1,829	2052	15.8
No education	4182	4143	50.9	7,852	7752	59.7
**Maternal occupation**						
Not working	1699	1924	23.6	9,996	9384	72.3
Working in agriculture	5520	5256	64.5	1,075	1531	11.8
Working in non-agriculture	977	968	11.9	1,959	2061	15.9
Missing	0	0	0.0	4	3	0.0
**Paternal educational status**						
Secondary and above	1878	1777	21.8	4,869	4333	33.4
Completed primary	3233	3186	39.1	3,179	3504	27.0
No education	3085	3184	39.1	4,976	5135	39.6
Missing	0	0	0.0	10	8	0.1
**Paternal occupation**						
Working in non-agriculture	5433	5379	66.0	10,517	10240	78.9
Working in agriculture	2543	2528	31.0	2,096	2418	18.6
Not working	0	0	0.0	417	318	2.5
Missing	220	240	3.0	4	3	0.0
**Pooled Household Wealth Index**						
Quintile 1 (Wealthiest)	1540	1554	19.1	2,487	2591	20.0
Quintile 2	1543	1642	20.2	2,409	2259	17.4
Quintile 3 (Middle)	1538	1598	19.6	2,516	2448	18.9
Quintile 4	1439	1413	17.4	2,553	2393	18.4
Quintile 5 (Poorest)	1649	1409	17.3	2,504	2569	19.8
Missing	487	531	6.5	565	719	5.5
**Maternal and newborn characteristics**						
**Maternal age at child birth**						
20 to 24 years	3097	3078	37.8	5,339	5372	41.4
Less than 20	4546	4509	55.4	5,453	5344	41.2
25 and more	553	560	6.9	2,242	2263	17.4
**Sex**						
Female	3833	3842	47.2	6,193	6117	47.1
Male	4363	4305	52.8	6,841	6862	52.9
**Birth rank and birth interval**						
2^nd^/3^rd^ birth rank, >2 yrs interval	2798	2794	34.3	2,820	2781	21.4
1^st^ birth rank	2334	2396	29.4	2,377	2383	18.4
2^nd^/3^rd^ birth rank, ≤2 yrs interval	853	863	10.6	1,711	1796	13.8
≥4^th^ birth rank, >2 yrs interval	1708	1620	19.9	4,279	4160	32.1
≥4^th^ birth rank, ≤2 yrs interval	503	475	5.8	1,847	1860	14.3
**Desire for pregnancy**						
Wanted then	5675	5687	69.8	10,161	10040	77.4
Wanted later	1058	1054	12.9	1,401	1420	10.9
Wanted no more	1463	1406	17.3	1,430	1468	11.3
Missing	0	0	0.0	42	51	0.4
**Perceived birth size**						
Average	5011	5040	61.9	7,721	7883	60.7
Smallest or smaller than average	1496	1377	16.9	3373	3294	25.4
Largest or larger than average	1682	1721	21.1	1883	1741	13.4
Missing	7	9	0.1	57	61	0.5
**Timing of initiation of breastfeeding**						
Never breastfed	94	102	1.3	640	714	5.5
<1 hours	5414	5196	63.8	5,513	4694	36.2
1 to 24 hours	1730	1721	21.1	3,632	3423	26.4
>24 hours	958	1129	13.9	3,228	4139	31.9
Missing	0	0	0.0	21	10	0.1
**Perinatal health services variables**						
**Number of antenatal care visits**						
No antenatal care visit	1757	1679	20.6	3,968	3821	29.4
<4 antenatal care visits	3102	3220	39.5	4,693	4860	37.4
≥4 antenatal care visits	3336	3248	39.9	4,346	4267	32.9
Missing	1	1	0.0	27	32	0.3
**Mode of delivery and delivery assistance**						
Traditional birth attendant/other untrained person	5743	5720	70.2	6,459	6540	50.4
Health professional with non-Caesarean section	2138	2087	25.6	5,172	4826	37.2
Health professional with Caesarean section	315	340	4.2	1,343	1552	12.0
Missing	0	0	0.0	60	62	0.5
**Place of delivery**						
Home	5688	5663	69.5	7,024	7089	54.6
Public hospital/clinic	1788	1746	21.4	2,118	1763	13.6
Private hospital/clinic	720	739	9.1	3,864	4091	31.5
Missing	0	0	0.0	28	36	0.3
**Average proportion of children vaccinated for BCG in each survey cluster** **	0.92 (±0.001)	0.92 (±0.006)		0.76 (±0.002)	0.78 (±0.008)	

† Unweighted.

†† Weighting was applied to compensate for the multistage cluster sampling design.

*included Gilgit and Islamabad regions.

**Mean (±SD)

BCG: Bacillus Calmette-Guerin. CI: Confidence interval.

NA: Not applicable.

NDHS: Nepal Demographic and Health Survey.

PDHS: Pakistan Demographic and Health Survey.

Table 2 shows the prevalence of IFA supplementation in Nepal and Pakistan. About 70% of Nepalese mothers and 44% of Pakistani mothers reported using any IFA supplements during their most recent pregnancy within 5 years prior to the interview. The mean (95% CI) number of IFA supplements used throughout pregnancy was 107 (95% CI = 105–109) supplements per pregnant woman in Nepal and 77 (95% CI = 75–79) supplements per pregnant woman in Pakistan. About one-third of Nepalese and nearly 9% of Pakistani mothers used >90 supplements throughout pregnancy. In Nepal, 58% of mothers and in Pakistan 31% of mothers started IFA at or before 5^th^ month of pregnancy. The combination of timing of start of IFA supplements with the total number of supplements used showed 33% of Nepalese mothers and 9% of Pakistani mothers started supplements at or before 5^th^ month of pregnancy with >90 supplements used throughout pregnancy.

**Table 2:** Prevalence of any iron-folic acid (IFA) supplementation, number of IFA supplements used, timing of start of IFA supplements and a combination of timing of start with number of IFA supplements used in Nepal and Pakistan.

	Nepal Pooled NDHS			Pakistan Pooled PDHS		
Variables	n†	n††	%††	n†	n††	%††
**Any IFA supplementation**						
No	2593	2481	30.5	7083	7232	55.7
Yes	5603	5666	69.6	5918	5707	44.0
Missing	0	0	0.0	33	41	0.3
**Number of IFA supplements used**						
Mean (95% CI)	5187	5233	107 (105–109)	4429	4311	77 (75–79)
Median (interquartile range)	5187	5233	120 (30–180)	4429	4311	60 (30–120)
**Number of IFA supplements used categories**						
None	2593	2481	30.5	7083	7232	55.7
1–90 supplements	2501	2561	31.4	3237	3204	24.7
>90 supplements	2686	2672	32.8	1192	1107	8.5
Missing	416	434	5.3	1522	1437	11.1
**Timing of start of IFA supplements**						
No IFA supplementation	2593	2481	30.5	7083	7232	55.7
At or before 5^th^ month of pregnancy	4755	4754	58.4	4318	4073	31.4
After 5^th^ month of pregnancy	732	784	9.6	1022	1052	8.1
Missing	116	128	1.6	611	623	4.8
**Timing of start and number of IFA supplements used**						
No IFA supplementation	2593	2481	30.5	7083	7232	55.7
At or before 5^th^ month of pregnancy & 1–90 supplements used	1894	1910	23.4	2517	2468	19.0
At or before 5^th^ month of pregnancy &>90 supplements used	2679	2665	32.7	1191	1106	8.5
After 5^th^ month & any IFA supplements used	614	657	8.1	721	737	5.7
Missing	416	434	5.3	1522	1437	11.1

† Unweighted.

†† Weighting was applied to compensate for the multistage cluster sampling design.

NDHS: Nepal Demographic and Health Survey.

PDHS: Pakistan Demographic and Health Survey.

Factors associated with early neonatal mortality in Nepal and Pakistan are presented in Table 3. In Nepal, maternal educational status, birth rank and birth interval, perceived birth size, sex of the baby, the timing of initiation of breastfeeding and the number of antenatal care visits were associated with early neonatal mortality. In Pakistan, maternal occupation, perceived birth size and the timing of initiation of breastfeeding were associated with early neonatal deaths.

**Table 3:** Adjusted hazard ratios (95% CI) for early neonatal mortality for community-level and socioeconomic, maternal and newborn characteristics and perinatal health service determinants in Nepal and Pakistan.

	Nepal Pooled NDHS		Pakistan Pooled PDHS	
Variables	aHR	95% CI	aHR	95% CI
**Maternal educational status**				
Secondary and above	1.00	(reference)	NS	
Completed primary	1.62	(0.86–3.07)		
No education	1.97	(1.09–3.49)		
**Maternal occupation**				
Not working	NS		1.00	(reference)
Working in agriculture			2.54	(1.91–3.36)
Working in non-agriculture			1.70	(1.25–2.32)
**Birth rank and birth interval**				
2^nd^/3^rd^ birth rank, >2 years interval	1.00	(reference)	NS	
1^st^ birth rank	2.63	(1.52–4.55)		
2^nd^/3^rd^ birth rank, ≤2 years interval	2.20	(1.31–3.72)		
≥4^th^ birth rank, >2 years interval	0.83	(0.40–1.76)		
≥4^th^ birth rank, ≤2 years interval	2.71	(1.55–4.74)		
**Perceived birth size**				
Average	1.00	(reference)	1.00	(reference)
Smallest or smaller than average	1.22	(0.77–1.91)	0.96	(0.72–1.28)
Largest or larger than average	1.93	(1.19–3.14)	1.45	(1.06–1.98)
**Sex of baby**				
Female	1.00	(reference)	NS	
Male	1.63	(1.03–2.60)		
**Timing of initiation of breastfeeding**				
Never breastfed	1.00	(reference)	1.00	(reference)
<1 hours	0.0046	(0.003–0.007)	0.0164	(0.011–0.024)
1 to 24 hours	0.0041	(0.002–0.009)	0.0104	(0.007–0.016)
>24 hours	0.0038	(0.001–0.010)	0.0072	(0.004–0.013)
**Number of antenatal care visits**				
No antenatal care visit	1.00	(reference)	NS	
<4 antenatal care visits	0.89	(0.57–1.39)		
≥4 antenatal care visits	0.45	(0.27–0.75)		

927 missing values were excluded from the pooled NDHS analysis and 696 missing values were excluded from the pooled PDHS analysis.

*Both models were adjusted for region/province and area of residence, maternal marital status, maternal level of attained education, maternal occupation, paternal level of attained education, paternal occupation, pooled household wealth index, maternal age at childbirth, child's sex, a combined variable birth rank and birth interval, maternal desire for the pregnancy, maternal perceived birth size, timing of initiation of breastfeeding, number of antenatal care visits, place of delivery, a combined variable for mode of delivery with delivery assistance, duration of recall period, year of birth of the child and the average cluster coverage of Bacillus Calmette-Guerin (BCG) vaccine.

aHR: Adjusted Hazard Ratio.

CI: Confidence Interval.

NDHS: Nepal Demographic and Health Survey.

NS: not significant.

PDHS: Pakistan Demographic and Health Survey.

The effect of any IFA supplementation on early neonatal deaths as determined by Cox proportional multivariate analysis in Nepal and Pakistan is shown in Table 4. The adjusted risk of early neonatal deaths was significantly reduced by 51% among Nepalese infants (aHR = 0.49, 95% CI = 0.32–0.75) and 23% among Pakistani infants (aHR = 0.77, 95% CI = 0.59–0.99) whose mother used any of IFA compared to no IFA supplements. Infants whose mother took >90 IFA supplements during pregnancy, the adjusted risk of early neonatal death was significantly reduced by 57% in Nepal (aHR = 0.43, 95% CI = 0.27–0.71) and 45% in Pakistan (aHR = 0.55, 95% CI = 0.33–0.90) compared to those whose mother did not take IFA. In Nepal, a significant reduction in the adjusted risk of early neonatal deaths of 45% were also observed in infants whose mother took 1–90 supplements compared to infants whose mother did not take IFA. With initiation of IFA at or before 5^th^ month of pregnancy, the adjusted risk of early neonatal deaths was significantly reduced by 53% in Nepal (aHR = 0.47, 95% CI = 0.31–0.73) and 28% in Pakistan (aHR = 0.72, 95% CI = 0.54–0.97) compared to no IFA. Infants whose mother started supplements at or before 5^th^ month of pregnancy and took >90 supplements had significantly lower adjusted risk of early neonatal deaths by 56% in Nepal (aHR = 0.44, 95% CI = 0.27–0.71) and 45% in Pakistan (aHR = 0.55, 95% CI = 0.33–0.90) compared to infants whose mother never used IFA during pregnancy. In Nepal, infants whose mother started supplements at or before 5^th^ month of pregnancy and took 1–90 supplements also had a significantly lower adjusted risk of early neonatal deaths by 44% compared to no IFA.

**Table 4:** Effect of any iron-folic acid (IFA) supplements, number of supplements used, timing of start of supplements and timing of start with number of supplements used on early neonatal mortality determined by multivariate analysis in Nepal and Pakistan.

	Nepal Pooled NDHS		Pakistan Pooled PDHS	
Variables	aHR	95% CI	aHR	95% CI
**Any IFA supplementation**				
No	1.00	(reference)	1.00	(reference)
Yes	0.49	(0.32–0.75)	0.77	(0.59–0.99)
**Number of IFA supplements used**				
No IFA supplementation	1.00	(reference)	1.00	(reference)
1–90 supplements used	0.55	(0.31–0.95)	0.88	(0.66–1.17)
>90 supplements used	0.44	(0.27–0.71)	0.55	(0.33–0.90)
**Timing of start of IFA supplements**				
No IFA supplementation	1.00	(reference)	1.00	(reference)
At or before 5^th^ month of pregnancy	0.47	(0.31–0.73)	0.72	(0.54–0.97)
After 5^th^ month of pregnancy	0.64	(0.28–1.48)	0.96	(0.77–1.48)
**Timing of start with number of IFA supplements used**				
No IFA supplementation	1.00	(reference)	1.00	(reference)
At or before 5^th^ month of pregnancy & 1–90 supplements used	0.56	(0.32–0.98)	0.87	(0.64–1.20)
At or before 5^th^ month of pregnancy &>90 supplements used	0.43	(0.27–0.73)	0.55	(0.33–0.90)
After 5^th^ month of pregnancy & any IFA supplements used	0.48	(0.18–1.28)	0.89	(0.51–1.56)

927 missing values were excluded from the pooled NDHS analysis and 696 missing values were excluded from the pooled PDHS analysis. Further, we excluded 416 and 1489 cases with implausible values of IFA supplements consumed from the analysis of number of IFA supplements used in NDHS and PDHS respectively.

*Both models were adjusted for region/province and area of residence, maternal marital status, maternal level of attained education, maternal occupation, paternal level of attained education, paternal occupation, pooled household wealth index, maternal age at childbirth, child's sex, a combined variable birth rank and birth interval, maternal desire for the pregnancy, maternal perceived birth size, timing of initiation of breastfeeding, place of delivery, a combined variable for mode of delivery with delivery assistance, duration of recall period, year of birth of the child and the average cluster coverage of Bacillus Calmette-Guerin (BCG) vaccine.

aHR: Adjusted Hazard Ratio.

CI: Confidence Interval.

NDHS: Nepal Demographic and Health Survey.

PDHS: Pakistan Demographic and Health Survey.

We also found that any IFA supplementation had a protective effect on neonatal deaths (0–30 days of life) only in Nepal. The adjusted risk of neonatal deaths was significantly lower in Nepalese infants whose mother reported use of any IFA supplements during pregnancy (aHR = 0.58, 95%CI = 0.35–0.94) compared to infants whose mother did not use IFA. In Pakistan, we found no significant reduction in the adjusted risk of neonatal mortality with the maternal use of IFA supplements during pregnancy compared to no IFA (aHR = 0.95, 95% CI = 0.74–1.23).

Our PAR estimates showed that 22.8% (95% CI: 0.5%–30.9%) of early neonatal deaths in Nepal and 36.4% (95% CI: 1.0%–60.8%) of early neonatal deaths in Pakistan were attributed to lack of starting supplements at or before the 5^th^ month of pregnancy and not taking >90 supplements. An estimated 4,600 deaths in Nepal and 75,000 deaths in Pakistan in early neonatal period could be averted each year if all pregnant women started IFA at or before the 5^th^ month of pregnancy and took >90 supplements throughout pregnancy.

## Discussion

### Main findings and their significance

Our study found that the degree of impact of antenatal IFA on early neonatal deaths was higher among Nepalese infants than Pakistani infants. The adjusted risk of early neonatal deaths was significantly reduced by 51% among Nepalese and 23% Pakistani infants whose mother used any IFA supplements during their most recent pregnancy within 5 years prior to interview. We found that most of this difference in the level of the mortality sparing effect between Nepal and Pakistan was due to the timing of the start of supplementation and the number of the supplements used. Among infants whose mother started supplements at or before 5^th^ month of pregnancy and took >90 IFA, the adjusted risk of early neonatal deaths was significantly reduced by 56% in Nepal and 45% in Pakistan. However number of supplements used analyses showed that taking >90 supplements accounted for most of change in the level of the mortality sparing infants compared to starting at or before 5^th^ month of pregnancy (see Table 4). With universal coverage of early initiation of IFA (at or before the 5^th^ month of pregnancy) and with use of >90 supplements, about 4,600 and 75,000 early neonatal deaths could be averted each year in Nepal and Pakistan, respectively.

The current study extends the findings of earlier studies from Indonesia and China which showed a protective effect of the use of IFA supplements during pregnancy on early neonatal deaths. [14], [16] A recent pooled analysis using three NDHS (2001, 2006 and 2011) has reported a significant reduction in the adjusted risk of early neonatal deaths by 45% (aHR = 0.55, 95% CI = 0.38–0.79) with any use of IFA supplements during pregnancy [31]. The current study findings provide important evidence to program mangers and policymakers to modify current IFA programs in Nepal and Pakistan and to encourage pregnant women to start IFA supplements earlier and to continue them throughout pregnancy.

### Strengths and limitations

The major strength of the study was the use of nationally representative samples from both countries. Both, the NDHS and the PDHS were conducted during the same time period, which allowed us to compare their findings by using similar multivariate analysis methods to adjust for potential confounding factors. Nevertheless, the existing IFA supplementation program has been modified in Nepal between 2003 and 2011 [13]. Both, the NDHS and PDHS used the same core questionnaires and the information collected for births and deaths in all surveys were of good quality as there were slight variations seen in the mortality rates for the overlapping time periods [9]–[12]. Moreover, the same organizations in Nepal and Pakistan conducted the repeated surveys in each country with robust quality control methods [9]–[12]. The recorded birth history information in DHS allowed us to identify cohorts of women from their most recent live birth five years prior to interview. Although, the information on exposures and outcomes was collected at the same time, an important element of timing (a retrospective measurement of the use of IFA supplements prior to birth) allowed causal inference. Our samples from both countries were large enough to allow us to analyse the effects of the timing of the start of supplementation and different number of IFA supplements used on the mortality outcome examined, while controlling for a range of potential confounding factors. We minimized the recall bias for the duration between the date of death of a child and date of interview, and use of IFA supplements during pregnancy by selecting the most recent singleton live births within 5 years prior to interview [19], [32], [33]. To minimize the recall bias for reporting the number of IFA supplements used, we excluded all those cases who reported a higher number of IFA used than maximum number could be taken when started from 5^th^ month or later in their pregnancy, assuming once daily use of IFA. We also adjusted our analysis for the number of times antenatal care services were used during the pregnancy [34].

The information on early neonatal mortality and use of IFA supplements could not be validated as it was based on maternal recall, which was the major limitation of the current study. However, mortality is a core measure in DHS and the methods for this outcome have been developed over the last 25 years [35]. Underestimation of early neonatal deaths could be possible in our sample as birth histories and child survival information were only collected from surviving mothers [19]. There is a possibility that maternal recall of the number of IFA supplements taken during pregnancy might not have been accurate and may have led to a misclassification of the women on the basis of categories of the number of IFA used. In addition, a heaping effect for the reported number of IFA supplements used was also observed in the data from both the countries. Furthermore, accessibility and quality of health services, environmental factors such as indoor air pollution, and presence of iron deficiency or iron deficiency anaemia during pregnancy which are also possible determinants of neonatal mortality were not available in the NDHS and the PDHS dataset. Some variables like maternal and paternal occupation which represented the employment status within the last 12 months preceding the survey, were not infant specific because these only presented the most recent conditions. Nevertheless, these limitations are important to discuss but we believe that these limitations are unlikely to have substantially influenced the validity of our study findings.

### Comparison with other studies

The protective effect of antenatal IFA supplementation on the risk of early neonatal deaths has been reported from China and Indonesia [14], [16]. A cluster randomised controlled trial from China found that the risk of early neonatal mortality in infants whose mother took IFA were significantly reduced by 54% (aHR = 0.46, 95% CI = 0.21–0.98) compared to those who took folic acid alone. Women in this trial started IFA supplements earlier in their pregnancy with an average gestational age at the time of start of supplements was 13.6 weeks. Moreover, a very high percentage of women took ≥120 supplements (84%) [14]. Post hoc secondary analyses of this trial have revealed that the neonatal mortality sparing effects were greatest in women who started IFA in the first trimester of pregnancy (71% reduction – aHR 0.29, 95%CI: 0.09–0.88) [36]. In this trial IFA also reduced the rate of preterm delivery and the neonatal deaths attributable to prematurity and birth asphyxia [14]. An observational study used data from three Indonesia DHS (1994, 1997 and 2002–03) reported a significant reduction in the risk of early neonatal deaths by 47% among children whose mother took any IFA compared to no IFA supplementation. Further, a significant reduction of 44% in the risk of early neonatal deaths was found in infants whose mother took ≥120 IFA during pregnancy. In Indonesia 22.5% of mothers took ≥90 supplements during their most recent pregnancy within 5 year prior to each survey [16]. Our previous pooled analyses of three NDHS (2001, 2006 and 2011) found a significant reduction by 45% (aHR = 0.55, 95% CI = 0.38–0.79) in the risk of early neonatal deaths with any use of IFA supplements compared to no use after adjusting for other confounding factors [31]. In the current study, in both countries, we have found the greatest mortality sparing effect on early neonatal deaths was with early initiation (at or before 5^th^ month) and use of a greater number of IFA. The finding is consistent with pooled analysis of Indonesia DHS study which found the greatest effect on under-five deaths (aHR = 0.44, 95% CI = 0.28–0.69) in infants whose mother started supplements in first trimester and took 120 or more IFA throughout pregnancy [15]. Our previous pooled analyses of three NDHS also found the greatest mortality sparing effect on neonatal deaths in infants whose mother started IFA in the first 4 months of pregnancy and took 150 or more IFA supplements throughout the pregnancy (aHR = 0.45, 95% CI = 0.24–0.85) compared to no use of supplements [31].

Both Nepal and Pakistan are using similar iron preparations and have almost identical rates of maternal anaemia [11], [37], however, the protective effect of maternally reported any use of IFA supplements on early neonatal deaths was lower in Pakistan compared to Nepal. The findings from our study suggest this difference could be due to the lower number of IFA supplements consumed by Pakistani women [mean IFA used were 77 (95% CI = 75–79) supplements per pregnant woman] compared to the Nepalese women [mean IFA used were 107 (95% CI = 105–109) supplements per pregnant woman]. To a lesser extent the lower percentage of Pakistani women (31%) who started supplements at or before 5^th^ month of their pregnancy compared to Nepalese women (58%) might have also contributed to the lower mortality sparing effect of use of IFA in Pakistan.

### Biological mechanisms and implications of study findings

The mechanism by which antenatal IFA supplementation reduces early neonatal deaths is unclear. The protective effect on early neonatal mortality could be due to a significant reduction in the risk of maternal anaemia with daily use of IFA [4], [7]. Anaemia during the first or second trimester of pregnancy is associated with higher risks of low birth weight, preterm delivery [4], and birth asphyxia [38], [39], and these are the major contributors of early neonatal mortality in low- and middle-income countries [3], [40], [41]. Therefore, prevention of these conditions could help reduce the number of deaths in the early neonatal period in these countries. The current approaches to prevent neonatal deaths, mainly secondary prevention interventions for delivery and postnatal care services, have had little effect on early neonatal deaths in high-mortality settings [2]. Therefore, countries like Pakistan and Nepal, where 80% of neonatal deaths occur in the first week of life, modification in their existing IFA program distribution strategies to initiate supplements early in pregnancy and improve compliance would help in reducing early neonatal mortality and subsequently contribute to achieving MDG-4 targets.

### Neonatal survival and program implications

Our PAR estimates used the mortality sparing effect for a sub-group analyses of the women who started IFA at or before the 5^th^ month of pregnancy and took >90 supplements throughout pregnancy. The sub-group analysis for PAR estimates has removed differences due to the effects of starting late and not taking at least 90 supplements. Based on our PAR calculations, about 23% and 36% of early neonatal deaths were attributed to not starting supplements earlier in pregnancy (at or before the 5^th^ month of pregnancy) and non-use of >90 IFA supplements in Nepal and Pakistan, respectively. With universal coverage of initiation of IFA at or before the 5^th^ month of pregnancy and use of >90 supplements throughout the pregnancy, we estimated that 4,600 early neonatal deaths in Nepal and 75,000 early neonatal deaths in Pakistan could be averted each year. Nepal in the recent past has strengthened its IFA supplementation program and modified the distribution strategy by involving female health care volunteers in rural areas. The enhanced program has increased the coverage of any IFA supplementation from 23% in 2001 [42]. to 80% in 2011 [11]. However, the coverage of any IFA supplementation and the number of supplements used showed variations in terms of area of residence and regions. On the other hand, in Pakistan the coverage of any IFA supplementation has remained static during the last decade (∼45%) and showed wide variations in coverage of IFA supplements by area of residence and provinces [9], [10]. In Pakistan IFA supplements are distributed through the static public health facilities and by lady health workers (community health workers). Pakistan needs to improve its existing IFA programs by adopting and implementing interventions similar to those employed by Nepal in the recent past [13]. At the same time, there is a need to enhance the existing IFA programs in both countries and encourage women to start supplements early and continue throughout their pregnancy. However, both countries will need additional resources to train health workers, to make IFA supplements free throughout pregnancy for all women, and to increase demand for antenatal IFA supplements through appropriate promotion programs.

To conclude, any IFA supplementation during pregnancy significantly reduced the adjusted risk of early neonatal mortality by 51% in Nepal and 23% in Pakistan. The greatest mortality sparing effect of IFA on early neonatal deaths was found in infants whose mother started the supplements at or before 5^th^ month of pregnancy and took >90 supplements throughout their pregnancy in both countries. These observational epidemiological findings need to be confirmed in a large sample, multicentre cluster randomized controlled trial using a stepped wedge design to explore the impact of early initiation and continuation of supplements throughout pregnancy on early neonatal mortality in these countries.
